# Case report: HSV lymphadenitis in immunocompromised patient with CLL

**DOI:** 10.3389/fonc.2024.1401208

**Published:** 2024-12-05

**Authors:** Talal Al-Assil, Shane Handelsman, Faisal Ansari, Ellen Flatley, Steven Stone, Mohammad Omaira

**Affiliations:** ^1^ Western Michigan University Homer Stryker M.D. School of Medicine, Kalamazoo, MI, United States; ^2^ Department of Medicine-Pediatrics, Western Michigan University Homer Stryker M.D. School of Medicine, Kalamazoo, MI, United States; ^3^ Department of Pathology, Western Michigan University Homer Stryker M.D. School of Medicine, Kalamazoo, MI, United States; ^4^ Pathology Services of Kalamazoo P.C., Kalamazoo, MI, United States; ^5^ Hematology & Medical Oncology, Bronson Cancer Center, Kalamazoo, MI, United States; ^6^ Department of Hematology-Oncology, Western Michigan University Homer Stryker M.D. School of Medicine, Kalamazoo, MI, United States

**Keywords:** Richter’s transformation, chronic lymphocytic leukemia (CLL), herpes virus (HSV), immune dysregulation, HSV lymphadenitis, CLL recurrence, reactivated dormant viral infections

## Abstract

**Background:**

Richter’s transformation (RT) in chronic lymphocytic leukemia (CLL) is associated with poor prognosis and requires prompt modifications in patient care. CLL patients are susceptible to severe infections due to immune dysregulation induced by their malignancy and immunosuppressive therapies.

**Case presentation:**

We present a case of a 63-year-old man with CLL who previously achieved remission and presented with a right inguinal mass. He was diagnosed with Rai Stage I CLL with del6q, without TP53 mutation, and treated with 6 cycles of fludarabine, cyclophosphamide, and rituximab (FCR) 6 years prior. Transformed CLL was suspected based on his lymphadenopathy, elevated lactate dehydrogenase, and constitutional symptoms, but excisional biopsy unexpectedly revealed herpes simplex virus (HSV)-1 and HSV-2, indicating a diagnosis of HSV lymphadenitis concurrent with CLL relapse with no transformation but acquisition of 17p deletion consistent with clonal evolution. The patient received three courses of dexamethasone and acyclovir, leading to successful clearance of the infection, evidenced by the resolution of his B symptoms. Subsequently, he was treated for the CLL recurrence with rituximab and venetoclax, demonstrating a favorable response with significant improvement in adenopathy and resolution of lymphocytosis.

**Discussion:**

This case highlights the possibility of reactivated dormant viral infections in the context of CLL relapse, underscoring the importance of comprehensive evaluation in CLL patients presenting with lymphadenopathy. Due to immunosuppressive defects and iatrogenic hypogammaglobulinemia, patients with CLL face an increased risk of viral infections, with HSV reactivation occurring more frequently and severely in the setting of hematologic malignancies and dysregulated T-cell immunity. Timely administration of antiviral therapy is crucial for HSV lymphadenitis to prevent rapid progression and debilitating symptoms. This case demonstrates the importance of considering atypical viral infection presentations in CLL patients and emphasizes the necessity of timely and adequate biopsies to differentiate between CLL transformation, HSV lymphadenopathy, and other causes of lymphadenopathy while avoiding unnecessarily aggressive lymphoma therapy.

## Introduction

The treatment options for chronic lymphocytic leukemia (CLL) have undergone significant advancements with the introduction of new targeted agents and monoclonal antibodies resulting in higher response rates (1). However, these therapies are associated with a higher degree of dysregulated immunosuppression, increasing patients’ susceptibility to major infections (2, 3). This includes the reactivation of dormant viral infections such as Epstein–Barr virus (EBV), varicella zoster (VZV), cytomegalovirus (CMV), herpes simplex (HSV), hepatitis B and C, and John Cunningham virus (JCV) (4). Among these, when reactivated, HSV can manifest as lymphadenitis, night sweats, and fever, which can resemble Richter’s transformation (RT).

RT describes the development of an aggressive large-cell lymphoma in the setting of an underlying small lymphocytic lymphoma (SLL) or CLL. Typically, diffuse large B-cell lymphoma (DLBCL) makes up approximately 90% of RT cases. Approximately 2%–10% of patients with CLL develop RT. These patients have poor prognoses (5). From the initial diagnosis of CLL to the development of RT, the median time frame is approximately 2 to 4 years (6). Typically, clinical features of RT are characterized by sudden clinical deterioration, painful lymphadenopathy, splenomegaly, and worsening “B” symptoms (i.e., night sweats, fever, and weight loss). Furthermore, the serum level of lactate dehydrogenase (LDH) is elevated in 50%–80% of cases.

HSV lymphadenitis in the context of CLL is very rare, and there have only been 19 cases reported in the literature published in the English language to date of this article (7–16). Reported cases range from concurrent diagnoses of CLL and HSV lymphadenitis to cases where the HSV infection develops decades after the initial CLL diagnosis. The development of HSV lymphadenitis does not appear to be associated with the amount of CLL treatment regimens that the patient has received. It has been observed in patients who have received multiple immunosuppressive regimens to no targeted leukemic therapy at all (13–19). However, treatment with fludarabine seems to be linked to increased susceptibility to herpes infections up to 17 months following completion of therapy (20). Due to the rarity of this disease, it is extremely important for clinicians to rule out reactivated dormant infections such as HSV lymphadenitis prior to exposing patients to possible treatment for RT. Herein, we present a case of a patient with CLL who appeared to have undergone RT but presented with an HSV lymphadenitis infection.

## Case description

A man in his 60s with a past medical history of ischemic left middle cerebral artery (MCA) stroke, seizures, and previously achieved CLL remission approximately 6 years prior presented to the clinic with a rapidly enlarging, painful right inguinal mass over 1 week. His initial diagnosis was with Rai Stage I CLL with 6q deletion, non-mutated TP53, and he was treated with 6 cycles of fludarabine, cyclophosphamide, and rituximab (FCR) regimen achieving complete clinical response. On physical examination, the mass was 7–8 cm in diameter and exquisitely tender. The rest of his exam was normal with no other lymphadenopathy. Laboratory results showed leukocytosis of 39,000 cells/μL with an absolute lymphocyte of 29,000 cells/μL (1,000–4,000 cells/μL) and an absolute monocyte of 5,600 cells/μL (200–1,000 cells/μL). The patient also exhibited mild normocytic anemia with a hemoglobin (Hb) of 13 g/dL (13.5–17.5 g/dL) and an elevated LDH of 366 IU/L (140–280). On a 2-week follow-up, his symptoms rapidly progressed to worsening lymphadenopathy, night sweats, lethargy, fever with a Tmax of 103°F, 15-pound weight loss, and worsening anemia to a Hb of 8.7 g/dL. This raised suspicion of CLL recurrence and RT.

**Figure 1:**
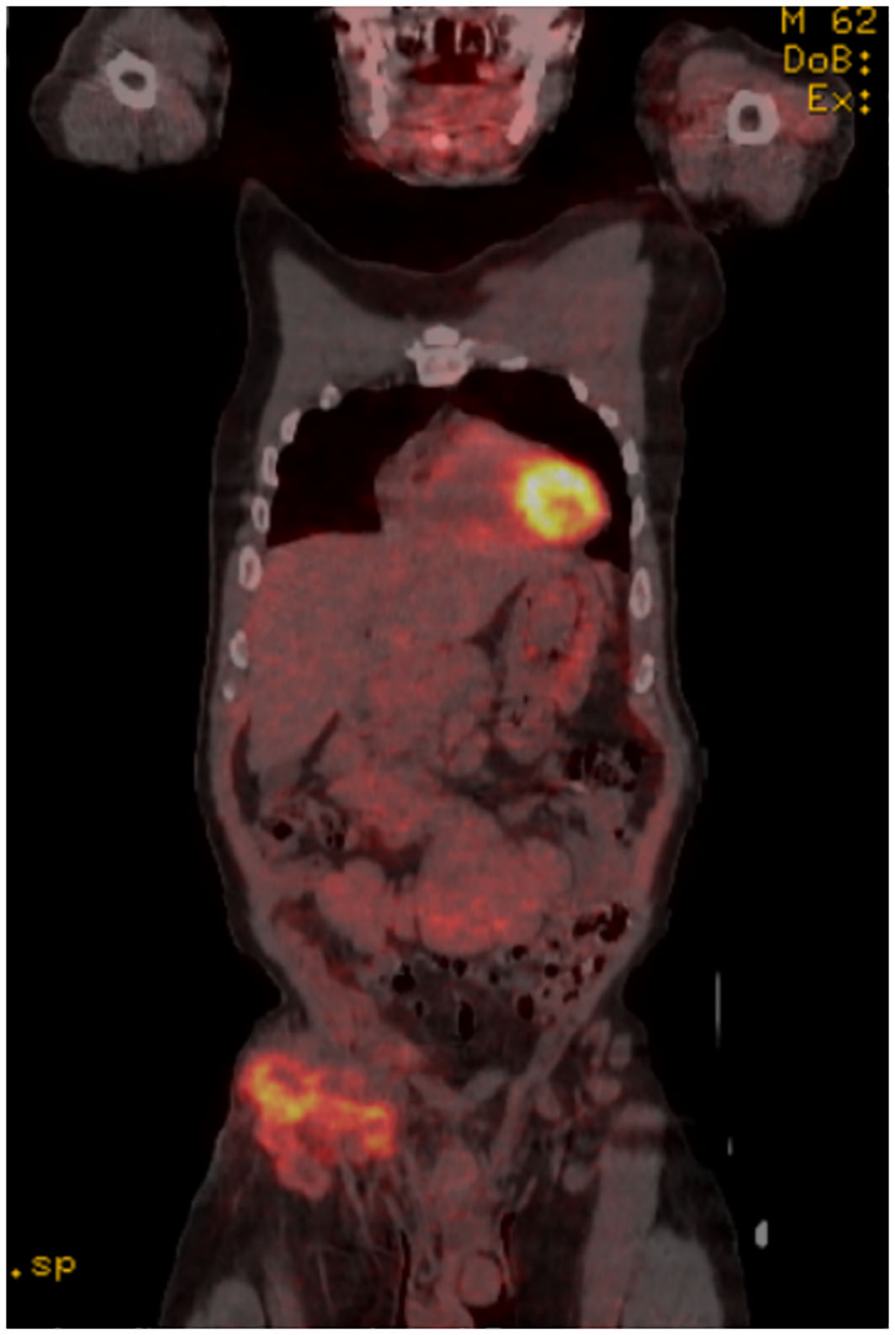
Coronal PET/CT scan showing high fluorodeoxyglucose (FDG) uptake in the R inguinal node.

**Figure 2:**
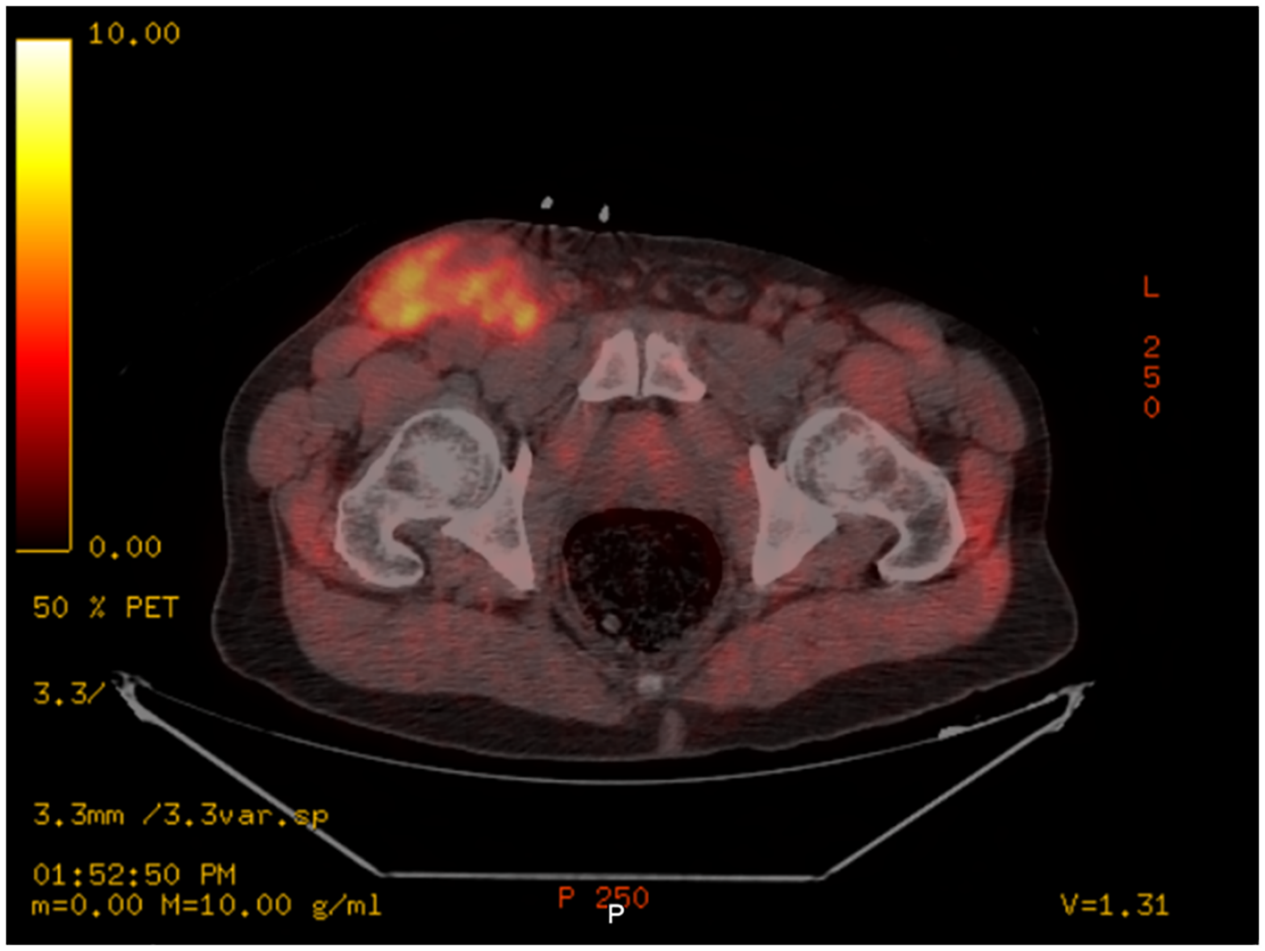
Axial PET/CT scan showing high fluorodeoxyglucose (FDG) uptake in the R inguinal node.

Positron emission tomography/computed tomography (PET/CT) revealed multiple bulky, mildly hypermetabolic lymphadenopathies with increased fluorodeoxyglucose (FDG) uptake in the right inguinal, iliac, retroperitoneal, and mesenteric regions, with a minimally hypermetabolic spleen and prominent axillary and subpectoral lymph nodes, suggesting recurrent lymphoma. Maximum standardized uptake value (SUV) was noted in the right inguinal mass, which measured 7.0 cm (**Figures 1**, **2**).

**Figure 3:**
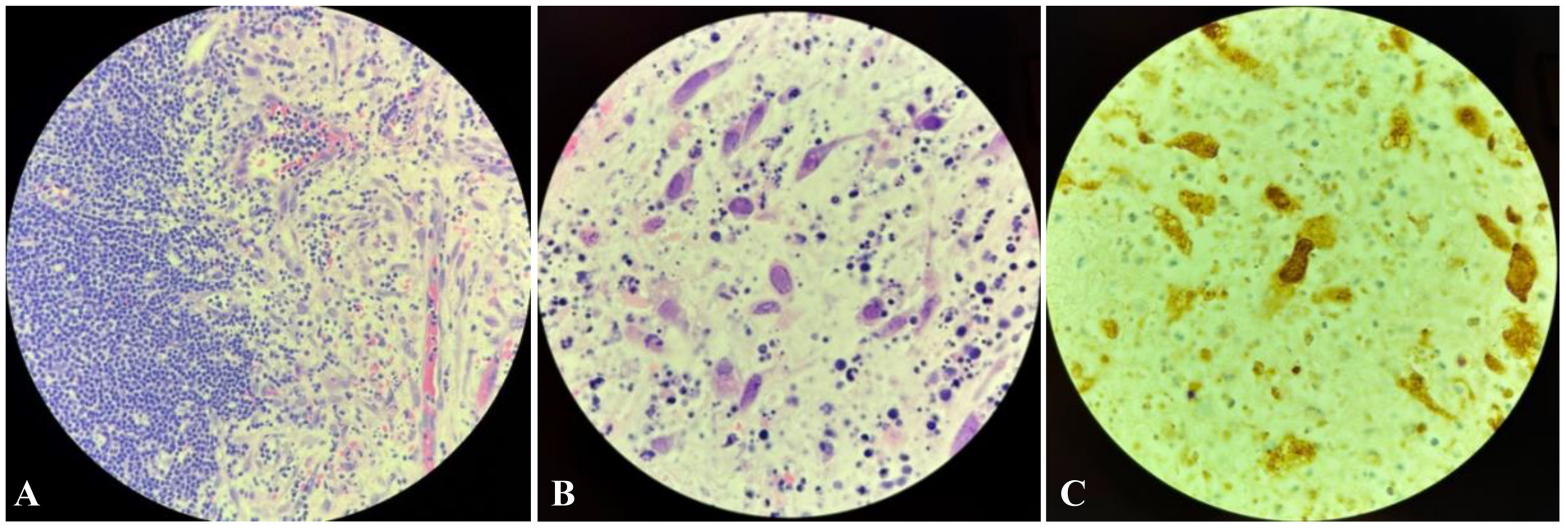
Inguinal excisional lymph node biopsy. **(A)** Hematoxylin and eosin (H&E) stain at ×400 magnification demonstrating lymphoid infiltration on the left side and stromal tissue with viral inclusions on the right. **(B)** H&E stain at ×1,000 oil immersion magnification showing necrotizing lymph node tissue with abnormally enlarged nuclei and margination of chromatin, suggestive of herpes simplex infection. A few eosinophilic inclusions raised the possibility of cytomegalovirus. **(C)** ×1,000 magnification with positive IHC staining for HSV-1 and HSV-2. Immunohistochemical (IHC) staining for cytomegalovirus (CMV) and Epstein–Barr virus [Epstein–Barr encoding region *in situ* hybridization (ISH)] were negative.

Given the patient’s history and presentation with new lymphadenopathy, B symptoms, and elevated LDH, relapsed CLL with RT was initially suspected. A core biopsy of the mass showed a small focus of viable small lymphocytes; however, this was inconclusive due to extensive necrosis. A subsequent excisional node biopsy of the right inguinal mass showed CD5+ B-cell lymphoid infiltration with small condensed monomorphic nuclei and scattered macrophages, along with stromal tissue with viral inclusions (**Figure 3**). CLL fluorescence *in situ* hybridization (FISH) studies revealed 17p deletion consistent with clonal evolution. These findings, along with positive immunostaining for HSV-1 and HSV-2, were consistent with a diagnosis of herpes lymphadenitis.

Prior to the initiation of HSV therapy, immunoglobulin levels were assessed. IgA and IgG were near the lower limit of normal at 67 mg/dL (69–309 mg/dL) and 607 mg/dL (613–1400 mg/dL), respectively, while IgM was within normal limits. Without the need for immunoglobulin supplements, the patient received antiviral therapy with acyclovir for 3 days and high-dose oral valacyclovir for 18 days to complete treatment for his HSV lymphadenitis. Long-term prophylactic valacyclovir twice daily was continued thereafter.

The patient’s symptoms improved remarkably with antivirals; however, his anemia and bulky lymphadenopathy persisted, warranting therapy for his relapsed CLL in the setting of the new 17p deletion. Per the Murano trial (21) and National Comprehensive Cancer Network (NCCN) guidelines, he was started on venetoclax and rituximab, leading to a complete clinical response, and then continued on maintenance venetoclax and valacyclovir for long-term prophylaxis.

## Discussion

In patients with CLL, RT presents with characteristic symptoms such as lymphadenopathy, elevated serum lactate dehydrogenase, and constitutional symptoms such as night sweats, fever, and unintentional weight loss (22). Although this transformation is rare, occurring in only approximately 2%–10% of all CLL cases, it is associated with a poor prognosis, with a median survival of 15 to 21 months after first-line treatment with R-CHOP (23).

Like in all cancers, immunosuppression is a common side effect. Immunosuppression in CLL can be attributed to both the immunosuppressive therapy used and the cancer itself, posing a significant risk for severe infection (24, 25). Specifically, following fludarabine-based chemotherapy, there is an increased risk for opportunistic infections due to a sustained drop in T-cell count for the first 2 years following completion of therapy (20). CLL itself also causes patients to exhibit imbalances in T-cell population subsets, including Th2 polarization and increased regulatory T cells (26). This skewed Th2 response comprises immunity against intracellular pathogens, instead favoring immunity against extracellular parasites. CLL also causes patients to be prone to various humoral defects, including hypogammaglobulinemia, IgA deficiency, and IgG subset deficiencies. Hypogammaglobulinemia, for instance, is thought to occur due to cellular interactions with malignant B cells and immune-inhibitory cytokines, resulting in reduced immunoglobulin production in a subset of CLL patients that is potentially irreversible (3). While hypogammaglobulinemia typically tends to make patients more vulnerable to bacterial infections, it does not exclude the possibility of severe viral infections. For example, several cases of patients with common variable immunodeficiency (CVID), a condition characterized by hypogammaglobulinemia, have been reported to have atypical viral infections, suggesting that a similar mechanism may be at play in CLL patients (27, 28).

Considering the presence of humoral defects and imbalance in T cells, it is understandable that CLL patients are susceptible to various atypical and potentially severe viral infections, including herpes viruses such as HSV, varicella zoster, and Epstein–Barr as well as hepatitis and JC viruses (4). Additionally, B cells in CLL have been found to have elevated levels of herpes virus entry mediator, which may render them particularly susceptible to HSV infection (29, 30). Typically, cases of latent HSV reactivation present with localized lymphadenopathy without systemic symptoms. However, cases involving hematologic malignancies and dysregulated T-cell immunity, as observed in CLL, will often experience more frequent and severe HSV recurrences (4, 28).

Importantly, the symptoms of HSV lymphadenitis can mimic those of RT in CLL, with ubiquitous lymphadenopathy and accompanying constitutional symptoms. Our patient highlights a case of HSV-associated lymphadenitis with symptoms mimicking RT. He presented with a bulky right inguinal swelling along with leukocytosis and elevated LDH. The condition progressed rapidly with fever, weight loss, severe anemia, and high levels of alkaline phosphatase. These findings raised our suspicion of a lymphomatous progression. Biopsy was positive for HSV-1 and HSV-2 immunostains yet negative for cyclin D1, lowering suspicion toward RT and increasing suspicion toward a diagnosis of HSV lymphadenitis. Concurrently, a PET/CT scan showed diffuse lymphadenopathy, and a FISH analysis of the patient’s peripheral blood revealed a 17p deletion-driven CLL clonal evolution.

With our patient’s unique presentation and the results of the inguinal mass excisional biopsy revealing an HSV infection, we hypothesize that the CLL relapse occurred first, followed by an HSV reactivation due to the state of immunosuppression that CLL patients exhibit. Upon discussing the results of the biopsy with the patient, he remarked that he had an HSV genital infection approximately 30 years prior. Interestingly, the CLL relapse was asymptomatic; rather, it was the symptomology of the HSV infection that allowed for an early detection of his relapse. In such cases, prompt antiviral therapy is crucial in the management of these patients to prevent rapid and potentially dangerous progression (21).

Despite the progression of CLL with 17p deletion and the emerging evidence favoring superior outcomes of indefinite Bruton’s tyrosine kinase inhibitor-based therapies, time-limited treatment with venetoclax and rituximab, as per the Murano trial, was selected (21). This approach not only favors shorter-time treatment but is also well endorsed by NCCN guidelines for patients with this molecular profile. Regarding our choice of antiviral prophylaxis, alternatives such as Shingrix and RZV vaccines continue to lack strong evidence in CLL patients. This is likely due to CLL patients’ suppressed immunogenicity and concern for the ability to mount an effective response from vaccines (31, 32). Ultimately, our patient achieved a complete clinical response and remained in remission and free from further viral reactivations for nearly 2 years.

These findings underscore the importance of carefully evaluating CLL patients who appear to be undergoing RT and performing adequate biopsies prior to starting possible treatment. If the diagnostic biopsy had not been conducted, the patient’s HSV reactivation would have been missed, and the patient may have been treated for CLL relapse, inadvertently exacerbating the concurrent HSV infection. Failure to diagnose HSV lymphadenitis early could lead to a misdiagnosis, a delay in treatment, and thus a detrimental impact on the patient’s outcome.

## Patient perspective

Due to the patient’s speech impairment secondary to stroke, he is unable to provide his direct perspective. Per his wife: “When his symptoms emerged, we were most concerned about a relapse of cancer, but when we went to his doctor, his levels were not indicating so. We were very confused. He was not able to communicate very effectively due to his speech impairment, but had he been able to express his genital sores, he could have been diagnosed sooner. His disability certainly delayed the diagnosis. Once he started the antivirals, his symptoms improved tremendously.” Signed informed consent is available upon request.

## Conclusion and learning points

- Our case underscores a potential association between CLL relapse and the occurrence of HSV lymphadenitis, but further research is required to establish a definitive link between the two.
- When assessing patients with lymphadenopathies, we advise maintaining a broad list of differential diagnoses. In this context, inquiring about a patient’s history of genital lesions becomes crucial, as these lesions could indicate the presence of dormant viruses such as HSV, HIV, VZV, HBV, or HCV. Given the immunocompromised state of CLL patients, there is a potential for reactivation of these viruses.
- This case presentation raises a pertinent question: should CLL patients be considered for antiviral prophylaxis without active disease during periods of observation?

## Data Availability

The original contributions presented in the study are included in the article/supplementary material. Further inquiries can be directed to the corresponding author.
